# Coeliac disease as a potential cause of idiopathic portal hypertension: a case report

**DOI:** 10.1093/gastro/gov065

**Published:** 2016-02-02

**Authors:** Saeid Yazdani, Ahmad Abdizadeh

**Affiliations:** Faculty of Medicine, Islamic Azad University, Tehran, Iran

**Keywords:** idiopathic portal hypertension, coeliac disease

## Abstract

Idiopathic portal hypertension is a disorder that has various clinical features. It is mostly characterized by bleeding oesophageal varices, obvious splenomegaly, anaemia and, occasionally, jaundice and ascites. Here we described an interesting case of idiopathic portal hypertension caused by coeliac disease in a 38-year-old woman. By putting this patient on a gluten-free diet, liver function tests became normal and portal vein diameter returned to normal range. This report indicates that, in coeliac disease, repetitive stimulation by antigens along the portal vein—and immune responses to them—can result in the development of idiopathic portal hypertension.

## Introduction

Coeliac disease is also known as coeliac sprue or gluten-sensitive enteropathy. Immune responses to gluten cause mucosal damage. Stimulation of the immune system is involved in pathogenesis of the disease. Non-cirrhotic (i.e. idiopathic) portal hypertension is a heterogeneous disorder with varying clinical features, and it is mainly demonstrated by signs and symptoms of portal hypertension in the absence of obvious hepatic disorder [1]. The aetiology of the disease is still unknown. There are various theories about the cause of this disease, such as autoimmunity disorder, chemical elements effect, infections, thrombosis, and hereditary causes [1, 2]. There are few cases of the co-existence of coeliac and hepatic disorders [3, 4]; here we report the interesting case of a 38-year-old woman with both idiopathic portal hypertension and coeliac disease.

## Case presentation

The patient was a 38-year-old married woman who had for several years been under observation because of ascites, splenomegaly and general weakness. She had been under treatment for possible cryptogenic cirrhosis and had received medication. When she was referred to our centre, we began our evaluation with a complete history of her existing and past problems and she mentioned several previous admissions for the purpose of evaluating her current problems. She had been taking Aldactone (spironolactone) for years. No history of hepatic or other gastrointestinal problems was found in her first-degree relatives. Her vital signs were normal and stable. She was mildly pale, with no sign of jaundice. Auscultation found lungs and heart normal. An obvious splenomegaly was detected in her abdomen. She had a mild oedema in the upper and lower limbs. We then requested laboratory tests: the results of autoimmunity tests, antinuclear antibody (ANA), anti-smooth muscle antibody (ASMA), and serum protein electrophoresis were reported to be normal. None of the laboratory findings indicated Wilson’s disease.

Ultrasonography of the abdomen showed heterogeneous changes of liver parenchyma, hepatosplenomegaly and high portal vein pressure (15 mmHg). Doppler ultrasonography of the portal vein then found no signs of thrombosis in the portal system. Ultrasonography had previously been carried out at other medical centres and backed up these findings. We carried out an upper gastrointestinal (GI) endoscopy and found grade 2 oesophageal varices. In accordance with our findings—and also according to clinical suspicion—we asked for a liver biopsy. The pathologic findings indicated mild chronic hepatitis, stage zero, grade 2, a hepatic activity index (HAI) score of 2 and no cirrhosis or malignancy.

**Figure 1.**
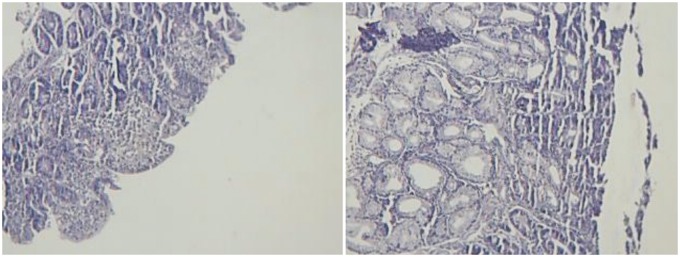
Duodenal biopsy shows flattening of mucosal villi with secretion of chronic inflammatory cells and neutrophils.

As our next step, serology tests for coeliac disease were requested. Anti-tissue transglutaminase (ATTG) was 18.8 (normal range <15), anti-endomesial antibody (AEA) was 27.8 (normal range <20). Histological findings of biopsy taken from duodenum showed flattening of mucosal villi with secretion of chronic inflammatory cells and neutrophils, equal to a Marsh score of 3 (Figure 1) and confirmed the presence of coeliac disease.

In accordance with these findings, a gluten-free diet was prescribed for the patient and after 3 months her general health was improved and the results of laboratory tests results returned to normal ranges. Also in ultrasonography work-ups, signs of portal hypertension were relieved and the diameters of the portal vein and common bile duct were 8 mm and 4 mm, respectively. Spleen size was normal and no ascites were detected. A serology test result showed a 50% decrease in AEA titration, although still not in normal range.

## Discussion

This has been a report of a case of coeliac disease presented by portal hypertension. Coeliac disease is intestinal dysfunction caused by gluten intolerance; the condition has various clinical presentations [5, 6]: in adults, the common symptoms are gastrointestinal disorders such as diarrhoea, fatty stool and weight loss, which are dependent on the severity of enteropathy. Other than the typical gastrointestinal presentations, coeliac disease has several extra-gastrointestinal presentations, e.g. neurological symptoms, ataxia [7], epilepsy [8], hepatobiliary disorders (PSC, autoimmune hepatitis) [3, 4, 9] and cirrhosis, dermopathy, infertility and anaemia [4, 10]; it is a little more common in women than in men. The prevalence of coeliac disease in the Middle East is similar to that in western countries [3, 11]. Work-up starts with clinical suspicion of the existence of coeliac disease [5, 6], after which serological tests will be requested, e.g. anti-endomesial antibody (AEA) and anti-tissue transglutaminase (ATTG). If the test results are positive, a biopsy of the small intestine is necessary to verify the diagnosis.

Idiopathic portal hypertension occurs secondary to microvascular occlusion of intra-hepatic small portal vein radicles. Multiple hypotheses regarding its pathogenesis have been proposed; one such entails the linking of gut disorders and subsequent hyper-coagulability in portal circulation. The role of a deficiency of ADAMTS 13 (a disintegrin and metalloproteinase with a thrombospondin type 1 motif, member 13), a von Willebrand factor-cleaving protease, is also being actively explored [12]. Ordinarily, in the first stages of disease, the liver function remains normal (normal albumin level and normal prothrombin time) but bleeding from oesophageal varices (92%), splenomegaly (96.7%), anaemia (26.2%) and hematemesis (23.7%) then occur [13]. Hepatic failure and its symptoms occur in the last stages of the disease. Other symptoms include ascites, hepatic encephalopathy and jaundice. The prognosis for the disease relies on its progression and is often good. A study made in Japan showed that, among persons affected by portal hypertension, the cause of 30% of deaths was bleeding from oesophageal varices, and that 25% were from hepatic failure [14].

The relationships of both primary biliary cirrhosis and autoimmune hepatitis to coeliac disease have been emphasized [15–17], but the relationship of coeliac disease to idiopathic portal hypertension has very rarely been pointed out in case reports [18–20]. In 2008, the Gastroenterology Center of Firuzgar Hospital (Tehran, Iran) also reported a case of coeliac disease with portal hypertension presentation, which was cured by gluten-free diet [20]. Some other reports have mentioned the concurrence of coeliac disease, splenic vein thrombosis and Budd-Chiari syndrome [21, 22]. The probable causes of non-cirrhotic portal hypertension in coeliacs are immune mechanisms stimulating immune mediators in the portal system [18, 19].

The concurrence of immune disorders and coeliac disease may account for the production of some immune complexes in the intestines of coeliac patients and would answer questions about variable presentations of this malabsorption syndrome. There are similar findings in idiopathic portal hypertension and coeliac disease which are caused by abnormal immune mediators (T-cells and some antibodies) [2]. In some past studies, repetitive antigen stimulations along the portal vein have been recognised as the possible cause of idiopathic portal hypertension [23].

Portal hypertension has traditionally been viewed as a progressive process, involving ultrastructural changes including fibrosis, nodule formation, and vascular thrombosis, leading to increased intrahepatic resistance to flow; however, it is increasingly recognized that a significant component of this vascular resistance results from a dynamic process, regulated by complex interactions between the injured hepatocyte, the sinusoidal endothelial cell, the Kupffer cell and the hepatic stellate cell, which impact on sinusoidal calibre [24].

The treatment of coeliac disease is based on eliminating gluten from the patient’s diet. The main sources of gluten are wheat, oats and secale. Gluten is also found in the capsules of medications [5, 25]. An untreated coeliac disorder results in lactase deficiency and intestinal epithelial cell damages so, to treat the disease, the patient should avoid using dairy and milk products [25]: many symptoms of the disease will be relieved by this dietary regimen. Our patient improved to a normal and healthy state after 3 months on a gluten-free diet. Laboratory findings and ultrasonographic studies showed that the portal hypertension disorder had been relieved.

The reporting of similar cases will increasingly clarify the relationship between coeliac disease and portal hypertension, leading to an aetiological approach to hepatic disorders and also coeliac disease in clinical work-ups.
